# Supplementary material for: A Critical View of the Application of the APEX Software (Aqueous Photochemistry of Environmentally-Occurring Xenobiotics) to Predict Photoreaction Kinetics in Surface Freshwaters
Source: Molecules. 2019 Dec 18;25(1):9. doi: 10.3390/molecules25010009 (PMC7017383; doi:10.3390/molecules25010009)
Supplement: Supplementary file 1 [file molecules-25-00009-s001.zip › 3.2.4_gcc-4.4.0.zip!/3.2.4_gcc-4.4.0/3.2.4_gcc-4.4.0/doc/octave/html/interpreter/Debugging.html]

Debugging - Untitled


Next: Input and Output,
Previous: Errors and Warnings,
Up: Top


---

## 13 Debugging

Octave includes a built-in debugger to aid in the development of
scripts. This can be used to interrupt the execution of an Octave script
at a certain point, or when certain conditions are met. Once execution
has stopped, and debug mode is entered, the symbol table at the point
where execution has stopped can be examined and modified to check for
errors.

The normal command-line editing and history functions are available in
debug mode.

- Entering Debug Mode- Leaving Debug Mode- Breakpoints- Debug Mode- Call Stack
